# “It's a life you're playing with”: A qualitative study on experiences of NHS maternity services among undocumented migrant women in England

**DOI:** 10.1016/j.socscimed.2020.113610

**Published:** 2021-02

**Authors:** Laura B. Nellums, Jaynaide Powis, Lucy Jones, Anna Miller, Kieran Rustage, Neal Russell, Jon S. Friedland, Sally Hargreaves

**Affiliations:** a Division of Epidemiology and Public Health, School of Medicine, University of Nottingham, Clinical Sciences Building, Hucknall Road, NG5 1PB, UK; b Institute for Infection and Immunity, St. George's, University of London, Cranmer Terrace, SW17 0RE, UK; c Infectious Diseases & Immunity, Imperial College London, Hammersmith Hospital, Du Cane Road, London, W12 ONN, UK; d Doctors of the World UK (Médecins du Monde), 29th Floor, One Canada Square, London, E14 5AA, UK

**Keywords:** Undocumented migrants, Refugees, Migrant health, Maternal health, Health inequalities

## Abstract

**Background:**

Undocumented migrant women experience complex barriers to maternity services, are less likely to receive the recommended level of maternity care, and have poorer obstetric outcomes than non-migrant women. There are concerns increasing restrictions on entitlement to health services have a detrimental impact on access to services and obstetric outcomes, particularly among undocumented migrant women. The study aimed to investigate the experiences of undocumented migrant women who have been pregnant in England, and factors affecting access to care and health outcomes.

**Methods:**

We conducted in-depth semi-structured interviews June–December 2017 with a purposive sample of migrant women born outside the UK (aged>18) who had experiences of pregnancy and undocumented status (without permission to reside) in the UK, recruited through Doctors of the World (DOTW) UK. Interpreting services were used on request. Interviews were recorded, transcribed, and analysed using thematic analysis. Ethical approval: Imperial College London Research Ethics Committee (ICREC reference: 17IC3924).

**Results:**

Semi-structured interviews were conducted with 20 participants, 10 of whom had their first antenatal appointment after the national target of 13 weeks, and nine of whom reported complications. Themes defining women's experiences of pregnancy included: restricted agency, intersecting stressors, and an ongoing cycle of precarity, defined by legal status, social isolation, and economic status.

**Conclusions:**

This study provides new evidence of women's experiences of pregnancy in the UK in the context of increasingly restrictive health policies including charging and data sharing. Six recommendations are made to ensure the UK and other migrant receiving countries work towards reducing inequalities and achieving national and global targets for maternal and child health and universal health coverage.

## Highlights

• Undocumented migrant women not entitled to free NHS England maternity services.
• Half of women interviewed experienced delays in accessing NHS maternity services.
• Key themes included restricted agency, stressors, and a cycle of precarity.
• Six immediate recommendations for policy and practice are drawn from the findings.

## 1 Introduction

There are clear calls to ensure the 250 million international migrants worldwide (WHO, 2018; UNHCR, 2015) are not ‘left behind’ (Winter 2019; Abubakar and Zumla, 2018). This has become underscored during the COVID-19 pandemic (Hargreaves et al., 2020). Nearly half of all migrants and refugees are women and girls (Women refugees), who are at increased risk of poor obstetric outcomes compared to non-migrants (Department of Health, 2015; Royal College of Obstetricians and Gynaecologists, 2008), and have been shown to seek antenatal care later, and access fewer maternity services (Heaman et al., 2013; Bollini et al., 2009; Almeida et al., 2013).

Approximately 14% of the UK population was born abroad, with an estimated 580,000 undocumented migrants (Prederi, 2013) - individuals without permission to reside including those who have been refused asylum, overstayed their visas, or entered the UK without permission (Office for National Statistics, 2011). In the UK, migrant women comprise 23% of maternal deaths (Mbrrace, 2015)*,* and are significantly less likely to receive the recommended level of antenatal care (Ameh and Van Den Broek, 2008; Lewis, 2007; Cantwell et al., 2011; Knight et al., 2015).

The increased risk of adverse obstetric outcomes among migrant women is attributable to social factors and barriers to timely and appropriate care (Royal College of Obstetricians and Gynaecologists, 2008; Bollini et al., 2009; Gagnon et al., 2009; Puthussery, 2016; Small et al., 2014; Jayaweera and Quigley, 2010; Rowe et al., 2008; Hacker et al., 2015), reflecting the concept of intersectionality, and the overlapping and interacting power structures, social identities, and inequities that define these women's experiences of care and health outcomes (Crenshaw, 2017). Undocumented migrant women also disproportionately experience risk factors such as destitution, violence, and exploitation, (de Jong et al., 2017; Bragg et al., 2019; Winters et al., 2018). In the UK, policies of deterrence, fear, lack of trust, language, cultural and system-level barriers, and inadequate information about where and how to access care have been highlighted as key challenges (Jayaweera and Quigley, 2010; Rowe et al., 2008; Henry et al., 2020; Winn et al., 2018; Floyd and Sakellariou, 2017). This has been reiterated in systematic reviews (Bollini et al., 2009; Gagnon et al., 2009; Small et al., 2014; Fair et al., 2020; Martinez et al., 2015; Woodward et al., 2014), and reflects evidence on the impact of exclusionary policies in other receiving countries (Campbell et al., 2014; Kuile et al., 2007; Martinez et al., 2015; Woodward et al., 2014). This reflects literature on ‘layers of disadvantage’ (Floyd and Sakellariou, 2017), the intersectionality women experience, and the compounded influences of power inequities, social, cultural, national, and ethnic identities, legal structures, and gender (Crenshaw, 1990). The numerous barriers to care experienced by migrant women during pregnancy further reflect the multiple levels in which Levesque et al.'s access to patient-centred healthcare model (Levesque et al., 2013) may be compromised (Henry et al., 2020).

Healthcare systems are devolved across the UK, with different systems in England, Scotland, and Wales. Whilst each country has a National Health Service (NHS), entitlement to free NHS services differs across the three countries.

Successive governments have implemented policies resulting in an increasingly restrictive health system for undocumented migrants. This has been criticised for neglecting the right to health or universal healthcare coverage (Borges and Guidi, 2018; Hiam et al., 2019). In 2004, a tightening up of the charging system for secondary care in England's National Health Service (NHS) took place, targeting individuals who are not ‘ordinarily resident’ in the UK, including undocumented migrants. The 2014 UK Immigration Act further restricted access to care, narrowing the definition of ‘ordinarily resident’, and introducing an immigration health surcharge for those applying for visas. The 2017 amendments to the Immigration Act made it obligatory for hospital trusts to identify whether individuals are chargeable for care, and to charge them upfront for the full cost of treatment prior to the delivery of any non-urgent care (Bragg et al., 2019). This upfront charging became mandatory, raising questions around healthcare providers' role as immigrant enforcement, and the alignment of these policies with core ethical and Hippocratic principles (Reynolds and Mitchell, 2019; Russell et al., 2019).

Separately, a Memorandum of Understanding between NHS England and the Home Office became public in 2017 and was suspended in 2018. This enabled patient data to be shared routinely for immigration enforcement purposes. To date, NHS Trusts are still required to inform the Home Office about patients who have had an outstanding debt of more than £500 to the NHS for over two months (Doctors of the World UK, 2017a). This information can then impact on future immigration applications. These changes reflect a wider focus across government policies to create a ‘hostile environment’ to deter immigration (Legido-Quigley et al., 2019).

In England, everyone is entitled to free primary care (Nellums et al., 2018a). However, undocumented migrant women are not entitled to free NHS maternity services, including antenatal, perinatal, or postnatal care, and are charged 150% of the NHS rate (Doctors of the World UK, 2017a; Citizens Advice, 2015). The cost of routine antenatal and postnatal care and an uncomplicated delivery starts at £6500 (Doctors of the World UK, 2017a). Whilst such care is deemed ‘urgent and immediately necessary’, and cannot be withheld if payment cannot be made upfront, women are still chargeable (Department of Health, 2016). However, there are significant inconsistencies in how chargeable patients are identified, notified, or charged across NHS Trusts, and a lack of transparency about charging policies (Feldman, 2017a). Furthermore, there is a lack of access to migrant health clinics or maternity services for undocumented patients within the NHS. As such, the charging regulations have made access to safe, appropriate, and timely care difficult for undocumented migrants (Royal College of Obstetricians and Gynaecologists, 2008), and do not align with England's commitment to reduce maternal mortality, stillbirth and neonatal death rates by 50% by 2030 (Knight, 2019).

Few studies have explored undocumented migrant women's experiences of pregnancy in England in the context of these health policies, or the layered and intersecting factors affecting access to and use of maternity services. Given high rates of maternal morbidity and mortality among migrant women in the UK, it is imperative we better understand their experiences to inform health policies that are not discriminatory and do not have a detrimental impact on health outcomes.

## 2 Methods

The aim of this qualitative study was to investigate the experiences of undocumented migrant women who have been pregnant whilst living in England in order to understand factors affecting access to care and maternal health outcomes. Ethical approval: Imperial College London Research Ethics Committee (ICREC reference: 17IC3924).

### 2.1 Study setting

We carried out this research with Doctors of the World (DOTW), an international human rights organisation focused on facilitating access to healthcare for excluded or marginalised communities. The research was conducted at the DOTW UK clinic in East London June–December 2017. The clinic does not perform deliveries, but provides support and advice on access to care, including through their Safe Surgeries initiative, which is targeted at tackling barriers to primary care registration for migrant populations in the UK (Bates, 2019). In 2019, Doctors of the World UK saw 3751 individuals, 98.8% of whom were migrants from outside of the European Union (EU) or European Economic Area (EEA) (DOTW, 2019). Approximately 56% were undocumented and without leave to remain in the UK, 89% had been unable to register with a GP despite being entitled to free primary care, 87% were below the poverty threshold, and 10% were vulnerably housed (Doctors of the World UK, 2017a). 86% of pregnant women who accessed the clinic had not accessed any antenatal care (Doctors of the World UK, 2017a).

### 2.2 Study participants and recruitment

The study included migrant women born outside the UK aged 18 or over who had experiences of pregnancy and undocumented status in England. We used purposive sampling to recruit a diverse group of participants representative of migrant women in the UK in relation to age, ethnicity, country of origin, and reason for migration (Office for National Statistics, 2011). We used snowball sampling to facilitate recruitment and invite women who may have experienced additional barriers to participation (Bryman, 2016; Braun and Clarke, 2006). We recruited women through posters, DOTW staff, DOTW women's and children's clinic records for the previous two years (which were only accessed by DOTW staff), and word of mouth through participants, DOTW volunteers, and local community organisations.

We provided potential participants a study information sheet, which we also read verbally, and women had the opportunity to ask questions before giving written informed consent. The study information clarified that the research was being carried out by the research team, and not Doctors of the World, and that electing or declining to participate would in no way affect their ability to access Doctors of the World, nor impact on their entitlement to NHS care or their immigration status. All information was translated or interpreted where preferred. Capacity to consent was assessed in line with the UK Mental Capacity Act Framework (Johnston and Liddle, 2007; Jones, 2005). Women deemed not to have capacity to consent, or who may have been put at risk by participating were not included. Participation was anonymous and confidential, and women could elect or decline to participate by contacting DOTW staff or the researchers. We offered participants reimbursement of £15 for their time, expertise, and travel costs.

### 2.3 Data collection and analysis

JP carried out semi-structured in-depth interviews using a piloted topic guide informed by evidence in this field, our previous research, and engagement with migrant communities. During piloting, we also engaged with participants to identify key topics they felt should be included, and strengthen acceptability. The researcher, who identifies as Black British, and comes from an academic background (Master's in Public Health), was not previously known to participants. The topic guide was structured using a narrative chronological approach (Groleau et al., 2006), and guided by methodology for research on sensitive topics and with culturally or linguistically diverse populations (Acker et al., 1983; DeVault, 1990; Renzetti and Lee, 1993; Racine, 2003; Rose, 2003; Telford and Faulkner, 2004; Gibbs, 2001; Beresford, 2005).

We conducted interviews in private rooms at DOTW. Interviews were approximately 1 hour. Where preferred, interviews were carried out with interpreters using Language Shop, a translation service routinely used by DOTW for simultaneous translation with professional trained medical interpreters targeted at providing interpreting services in healthcare contexts. Interviews were conducted in English, Albanian (n = 1), Bengali (n = 1), Mandarin (n = 1), and Punjabi (n = 1), and audio recorded with participants’ consent. The English language content of recorded interviews was transcribed verbatim by a professional transcription service and analysed in English.

We analysed transcripts in Nvivo 11.0 using thematic analysis, which enabled an in-depth exploration of women's experiences, and is appropriate for research with culturally and linguistically diverse participants (Bryman, 2016; Braun and Clarke, 2006; Squires, 2009). Recruitment was guided by saturation (Guest et al., 2006). Analysis followed the stages outlined by Braun and Clarke (2006). We adopted an inductive empiricist approach given the lack of previous empirical research on undocumented migrant women's experiences of maternity services in NHS England, as well as the policy changes relating to data sharing and charging that were implemented immediately preceding the research on which no data had yet been generated. Analysis was also informed by concepts of intersectionality (Crenshaw, 1990) and access to patient-centred healthcare (Levesque et al., 2013). Quality and rigour were strengthened using evaluative criteria for qualitative research (Cohen and Crabtree, 2008). Analysis was carried out by three researchers, and codes and themes were developed through discussion and an active process of reflexivity.

## 3 Results

### 3.1 Participant characteristics

We conducted 20 semi-structured interviews June–December 2017 (Table 1). Over half of participants were between 31 and 40 years. 17 women came to the UK to study or visit friends and family, and overstayed their visas. One woman was not undocumented at the time of interview, but previously experienced undocumented status, and two had been trafficked/smuggled into the UK. The majority of women were born in Africa (n = 7) or Asia (n = 8), and 13 did not speak English as a first language. 16 women had 1-2 children (including current pregnancies); 13 were pregnant at the time of interview.

**Table 1:** Characteristics of participants.

Characteristics	Participants N (%)
Total sample	20 (100.0%)
**Age**	
21–30	8 (40.0%)
31–40	11 (55.0%)
41–50	1 (5.0%)
**Immigration status at time of pregnancy**	
Visa overstayer	17 (85.0%)
Trafficked/smuggled	2 (10.0%)
Asylum seeker	1 (5.0%)
**Continent of birth**	
Africa	7 (35.0%)
Asia	8 (40.0%)
Europe	2 (10.0%)
North America	3 (15.0%)
**First language**	
English	6 (30.0%)
Non-UK	13 (65.0%)
Unknown	1 (5.0%)
**Years living in UK**	
0–5	10 (50.0%)
6–10	5 (25.0%)
11–15	3 (15.0%)
16 +	2 (10.0%)
**Parity (including current pregnancy)**	
1–2	16 (80.0%)
3–4	2 (10.0%)
5 +	2 (10.0%)
**Weeks at first presentation to antenatal care**	
Median (range)	12.5 (4–40)
Before 13 weeks	10
After 13 weeks	10
**Charges for care**	
Median (range)	£5327 (£3072-£11,500)
**Ability to pay for charges**	
Yes	3
No	17

10 women experienced delays in accessing antenatal care, presenting late to the NHS for their first antenatal appointment (median 12.5 weeks; range 4–40 (no antenatal care)). The national target is first antenatal appointment before 13 weeks of the pregnancy, although ideal presentation is 10 weeks (Royal College of Obstetricians and Gynaecologists, 2008). One woman presented to services for the first time at delivery, and had no antenatal appointments prior to that. Nine women reported complications during pregnancy, delivery, and postnatally, including premature birth and unexpected caesareans. Participants described being invoiced fees for NHS England maternity care ranging from £3072 to £11,500, with a median bill of £5372. 17 women reported significant financial difficulties and being unable to pay the charges.

### 3.2 Thematic analysis

**Fig. 1:**
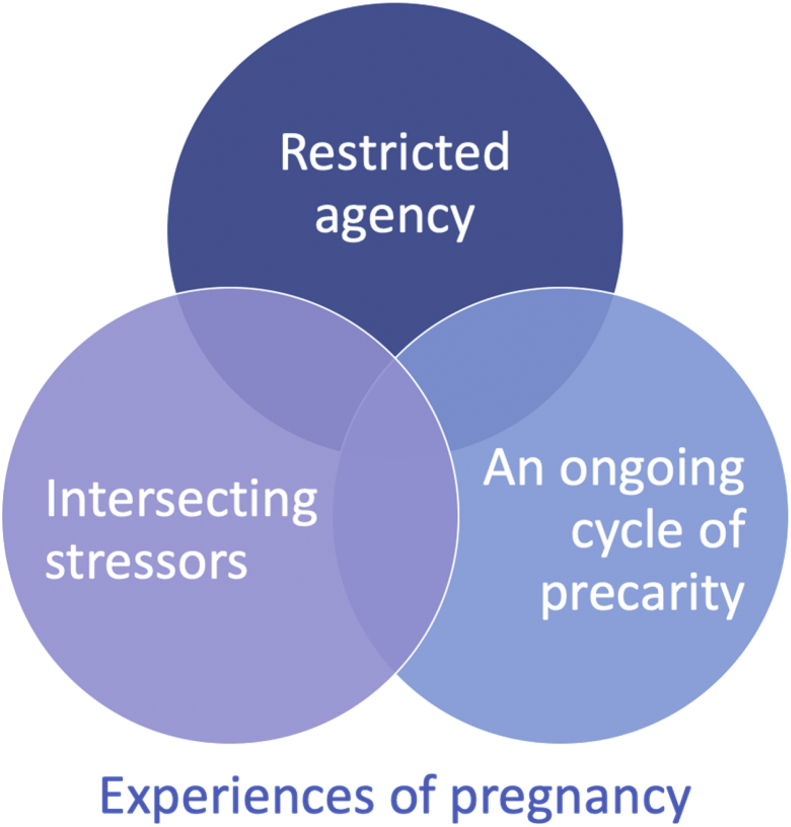
Key themes.

Women's experiences were defined by a dynamic experience of intersectionality, which reimagines static conceptualisations of this framework. Within this overarching framework, we identified key themes describing women's experiences of pregnancy (Fig. 1).

#### 3.2.1 Women's experiences of pregnancy

We identified three interrelated themes describing women's experiences of pregnancy and NHS maternity services in England, illustrating a dynamic experience of intersectionality. The first theme *restricted agency* describes the importance of agency – the ability to exert power or control, and the challenges women experienced when they felt they were not in control of their health or access to care. The second theme *intersecting stressors* captures the layered, interconnected, and interacting forces influencing women's access to patient-centred health care. Women's experiences illustrated by this theme were defined by their migrant status, linguistic background, socio-economic status, country of origin, and ethnicity, and the multiple stressors they experienced as a result of these factors, impacting on their health and well-being. The third theme *a cycle of ongoing precarity* describes the insecurity women experienced, defined by their legal status, social isolation, and economic status. This theme reflects the systemic and structural forces women experienced, which contributed to feelings of constantly being on the edge or at risk, which was difficult to escape because of the interconnectedness of these multiple structures creating precarity (Fig. 1).

### 3.3 Restricted agency

This theme was defined by women's undocumented status, inability to access care, and charges for routine maternity care, and the process of having power or control over their health, bodies, and lives restricted. As one woman poignantly expressed, highlighting the power and control of the health and immigration systems over both them and their unborn children:

“It's a life you're playing with.” [Interview 1]

Women's knowledge about their legal status and entitlement to healthcare was a salient example of where they felt they had limited power because they did not know what they could access or how to get the care they needed.

These experiences illustrated the power hierarchies situated in how knowledge is shared and accessed, and the interrelationship of knowledge and agency.

“I didn't know how to access health services because I wasn't sure because of my status in the country.” [Interview 2]

Women also felt powerlessness because they had not known that they would be charged. As a result, they could not make informed decisions.

“Just last month I've just received a bill…£5654…we don't have any income, nothing…No one told me that, ‘We are charging you for that’.’” [Interview 4]

Women emphasised the importance of better informing women upfront about their healthcare entitlements, and when they would be charged. There were significant inconsistencies in when and how women were informed they would be charged. Two women were told before receiving care that they would be charged, one of whom thought she could not access care until she could pay. The other women reported that they were not informed until after accessing care, either by health service staff, or by mail with a bill.

“I think it's better to know in advance, at least you prepare because when you're giving birth or just gave birth, everything is upside down, it's stressful, especially if you're a first mum, you don't know anything, so it's best to know.” [Interview 9]

The lack of transparency in provision of accessible and timely information to women about charging, data sharing, and entitlements to care created an environment through which structural power hierarchies restricted women's agency.

### 3.4 Intersecting stressors

Women experienced multiple stressors defined by the complex intersection of their immigration, financial, and health status.

“How can someone be pregnant with no job, no place to live, no food and you are sick, you don't even have access to go to the hospital.” [Interview 7]

Women described how the stressors associated with their pregnancies and financial challenges, including difficulties paying for housing, food, transportation, and medication all overlapped.

“I was suffering too much…I was sleeping rough outside…When I went [to the hospital] five months pregnant, they were asking me, ‘Have you got any scan?’ I said, ‘No.’…I couldn't manage anything. You're striving to get where you sleep, you don't know how you're going to eat.” [Interview 14]

The financial stressors women experienced also intersected with their immigration status. Because women were undocumented, they were both chargeable and not legally allowed to work.

“I was so scared…I don't have any status…I'm not working, I didn't even have a place on my own and I said, ‘How am I going to have a child now?’.” [Interview 7]

As a result, women experienced a ‘triple jeopardy’ defined by multiple socio-economic stressors such as vulnerable housing and destitution, significant charges for their pregnancy, and an inability to legally acquire the resources to pay their NHS bills or improve their financial situation. In some cases, this also impacted on how they were treated.

“One midwife…she was rude to me, said, ‘Hey, why don't you go back where you came from?’… I started crying because it was hurting me, tears came out of my eyes, I said ‘I can't go back, I'm so sorry for that, I can't’… she said, ‘You [go] back to where you come from, why don't you go?’... The money, we can't afford the money. At that time she said shouting at me, why we make the baby…because we didn't know about the money coming.” [Interview 14]

### 3.5 An ongoing cycle of precarity

The precarity women experienced was defined by the insecurity associated with lack of documentation, financial status, or health, which interacted to create an ongoing cycle.

Women described how the uncertainty (and lack of control) they had around their legal status, and whether or not they, their families, or their baby would be allowed to stay in the UK contributed to feelings of stress and fear during their pregnancy. This was exacerbated by and further contributed to financial stressors, and barriers to healthcare.

“[The hospital] turn you away…because your paper is not up to date…and the GP do the same thing, just the same also, they say ‘Come back again,’ and when you go back, they find a different excuse they give you.” [Interview 5]

A defining aspect of the precarity in women's situations was the experience of being without documents to enable them to access housing, work, a bank account, or healthcare.

“I found it very difficult having to know that I'm pregnant, I am destitute, I have no money and no home, and then I'm going to have to pay this bill.” [Interview 2]

Ultimately, women's narratives illustrated an ongoing cycle of precarity: women's immigration status prevented them from working and made them chargeable for care. This in turn deterred them from care, impacting on their health outcomes. Finally, incurring a debt to the NHS could lead to data sharing with the Home Office, and impact on future immigration applications or their ability to legally reside in the country. As one participant described, speaking about her own experiences and those of other undocumented migrant women:

“We can't work but we have to pay a bill, so you expect us to pay a bill if we can't work, you know?..They tell you if you don't pay, it can hamper your chances with Home Office.” [Interview 1]

## 4 Discussion

Women's experiences of maternity services were defined by *restricted agency*, *intersecting stressors*, and *an ongoing cycle of precarity.* These findings are consistent with the limited body of evidence on undocumented migrant women in the UK, which shows a detrimental and deterrent effect of charging and data sharing on women's use of maternity services and their health outcomes (Bragg et al., 2019; Nellums et al., 2018a; Alliance, 2019; Feldman, 2016, 2017b; Shortall et al., 2015). They also echo research with undocumented migrant women in other countries, showing these populations experience more unintended pregnancies, delays in accessing care, poor access to or uptake of screenings (e.g. cervical, infectious diseases), increased violence, a greater risk of sexually transmitted infections, low vaccination status including for rubella, and higher rates of pregnancy related risk factors (Wolff et al., 2005, 2008; Wendland et al., 2016; Reed et al., 2005; Munro et al., 2013).

Against the national target for pregnant women to have their first antenatal appointment before 13 weeks (Royal College of Obstetricians and Gynaecologists, 2008), it was found that half of the women interviewed (Bollini et al., 2009) experienced delays in accessing antenatal care, with a median time to first antenatal appointment of 12.5 weeks (range 4–40 weeks). This is compared to 96% of women who were seen before 12 weeks in the UK's 2014 national survey data (Redshaw and Henderson, 2014). As a result of these restrictive and deterrent policies, these births, which are recorded and registered, limit progress towards national targets to increase adequate provision of antenatal care, and decrease perinatal morbidity and mortality.

The findings reflect a recent study on the impact of NHS charging for maternity care on midwives, which showed delays in accessing care, discriminatory applications of the regulations, insufficient provision of information about entitlement to maternity care for women or providers, and the ongoing cycle of precarity perpetuated and exacerbated by charging and data sharing policies (Bragg et al., 2019). This aligns with a wider body of evidence demonstrating that even when individuals are entitled to care, barriers such as insecure legal status and marginalisation, misinformation, and hostile healthcare or social environments limit timely health seeking and can be structurally discriminatory (McLaughlin and Hennebry, 2013; Chase et al., 2017; Came, 2014). There is also evidence that charging and data sharing are impacting on other patient groups from migrant or ethnic minority backgrounds in addition to undocumented migrants, which may be attributed to the complexity of the regulations, poor provision or lack of proactive dissemination of data on entitlements, and inconsistencies in how charging is implemented (Psarros, 2018).

This study brings attention to the inconsistent and inaccurate application of charging regulations, which increases the risk of inequitable and discriminatory delivery of services, and avoidable barriers to urgent and immediately necessary care. Inappropriate implementation of the charging regulations has also been demonstrated in other research (Bragg et al., 2019; Shortall et al., 2015; Nellums et al., 2018b; Doctors of the World UK, 2017b; Hatherall et al., 2016; Poduval et al., 2015; Creative Research, 2013), despite Department of Health guidelines emphasising the importance of informing women they are chargeable, and consequences of unpaid debt (Overseas chargeable patients, 2019).

This underscores these inconsistencies are unlikely to be singular instances. In research commissioned by the Department of Health, it was estimated that entitlement to care was inaccurately assessed by Trusts for as many as 30% of patients, resulting in inappropriate charging (Creative Research, 2013). This is due to variations in what level of documentation is requested from individuals seeking care and which individuals are asked to provide evidence, differences across trusts in relation to how chargeable status is assessed and charges recouped, and significant inconsistencies in staff knowledge around charging policies and entitlement to care. This highlights the need to address systemic and structural inequities in how charging is implemented and the discriminatory effects this may have, as well as the need to proactively and meaningfully implement better training of staff at all levels relating to regulations around entitlement (including both charging and data sharing).

The findings illustrate that undocumented migrant women remain concerned about the confidentiality of their data, with fears that accessing health services may lead to immigration enforcement consequences. The impact of actual and perceived data sharing has been identified as a key barrier to care in other research, including both prior to and following the Memorandum of Understanding enabling data sharing between NHS Digital and the Home Office, which still occurs where a debt of £500 or more is unpaid for more than two months (Nellums et al., 2018b; Observatory Report, 2019; Chauvin et al., 2009; Dartnall et al., 2005).

There is evidence that some groups of women who are born outside the UK have elevated rates of avoidable adverse obstetric outcomes compared to women born in the UK (Lewis, 2007; Lewis and Drife, 2004). Furthermore, data clearly demonstrate that maternal mortality is frequently attributed to underlying conditions that could have been identified in antenatal care (Ameh and Van Den Broek, 2008; Lewis, 2007; Cantwell et al., 2011; Knight et al., 2015). The findings are also consistent with previous research demonstrating women from ethnic minority and migrant groups are significantly more likely to experience delays in accessing maternity care compared to white and UK-born women (Rowe et al., 2008; Cresswell et al., 2013), and evidence that nearly 50% of undocumented migrant women in the EU may not have any antenatal care during pregnancy (Chauvin et al., 2009).

The findings underscore the persisting barriers to care and inequalities undocumented migrant women in the UK experience. It is clear that ensuring equitable, timely, non-discriminatory, and compassionate care for this group is complex. However, the findings in this study provide poignant and compelling evidence that there is a need to facilitate – not restrict – access to maternity services in order to address the continuing high rates of maternal and child morbidity and mortality seen in the UK, particularly among ethnic minority and migrant women.

### 4.1 Theoretical contribution

The findings build on the framework of intersectionality, which is rooted in Black feminism and Critical Race Theory, highlighting the experience of women from diverse backgrounds, and particularly intersecting legal and social power structures, violence, and minoritisation, including for women from immigrant backgrounds (Crenshaw, 1990; Carbado et al., 2013). This research expands the interdisciplinarity of this theory in two key ways.

First, the findings emphasise the dynamic rather than static quality of intersectionality, and widen the experiences and structures of power this framework engages, including not only language, socio-economic status, and ethnicity, but also shifting immigration and legal statuses. This adds granularity to theoretical understandings of how these factors influence the dynamic structures of power defining marginalisation, including restricted agency, intersecting stressors, and ongoing cycles of precarity.

Second, the research pushes forward this framework by elucidating the interrelationship between two structures – the healthcare system and the immigration system. The former – in this case the National Health Service – is an organisation intended to improve health and well-being, guided by seven core principles (The NHS Constitution for England, 2015). The latter, however, has been deliberately molded as part of a system intended to create a ‘hostile environment’ with a ‘deterrent effect’ (Somerville et al., 2009). As such, the relationship between these structures shifts in line with immigration and health policies. Furthermore, the intersection of these two structures ultimately conflicted with the values of patient-centred care (Henry et al., 2020; Rafighi et al., 2016), and compounded existing factors limiting women's access to care, exacerbating structural inequities driving health disparities among migrant women.

These understandings of intersectionality in the narratives are illustrated in the interrelationship between the three core themes, with the power hierarchies in the health system and legal structures reaching beyond the *intersecting stressors* women described, inherent in the production of both *restricted agency* and *cycles of ongoing precarity*. Thus, women's experiences of intersectionality move beyond structural power inequities driven by ethnicity or race and gender, reflecting added structural and systemic marginalisation driven by diverse national, linguistic, economic, and legal identities. The findings illustrate why intersectionality is a dynamic framework that must be engaged and re-imagined as social and structural identities (and indeed agency and precarity) are re-envisioned in relation to changes in health, immigration, and other policies. Rather than a static theory, this research underscores why understandings of intersectionality must be conceptualised as an imperative for social change (Carbado et al., 2013).

### 4.2 Implications for policy and practice

The increasing restrictions on access to care for undocumented migrants have been strongly criticised by health professionals because of the risk they present for delaying and deterring access to health services, the potential inequities that may result in the application of the regulations, and the inappropriate overlap between health services and immigration enforcement (Russell et al., 2019; Smith and Dexter, 2018; Hiam and McKee, 2017; Hargreaves et al., 2016). The findings provide critical evidence that preventable complications are occurring. Beyond this, the findings flag that the impacts of the charging and data sharing policies align with the wider aim to create a hostile environment, which is structured to have a deterrent effect, and more broadly, the reluctance evident in the UK's approach to migrants (Somerville et al., 2009). Whilst there has been a government commissioned review to examine the impact of the 2017 amendments, and a review by Public Health England to examine the impact of the data sharing agreement, the findings from these reviews have not been published (O’Donnell et al., 2019). As a result, there have been calls by medical royal colleges, and the parliamentary health and social care select committee for more transparency about the findings, and the risk of a detrimental and inequitable impact on access to care and health outcomes (NHS, 2019; Correspondence with Secretary, 2019). Efforts should also be made to evaluate whether maternity services are migrant friendly, and the extent to which care provided is adequate and acceptable (Gagnon et al., 2014).

A key finding with immediate implications for changes in practice are the barriers women experienced in registering with a GP. These findings unfortunately reflect other evidence in this field, including recent research by DOTW UK demonstrating that among their clients, one in five requests to register with a GP are wrongly refused (Doctors of the World UK, 2017b). Guidelines clearly state that anyone, regardless of migrant status, is entitled to register with a GP, and there is no regulatory requirement to show proof of address, immigration status, or identity to register (NHS England, 2015). However, all but four of the participants had been unable to register with a GP prior to accessing DOTW. This study provides evidence that women are regularly being turned away from practices, and that registration is being refused.

In light of evidence of errors in the application of the charging regulations, there have been calls to strengthen training and provision of information for healthcare professionals (Torjesen, 2019). There is an urgent need to ensure staff in general practices are better informed about entitlements to primary care, and that training is provided to ensure individuals are not prevented from registering for or accessing primary care (Bragg et al., 2019; Torjesen, 2019). There have been successful models facilitating access to primary care for migrant populations such as the Safe Surgeries initiative, which can be used as examples of best practice (Bates, 2019).

However, this is secondary in importance to wider policies of deterrence, and the need to challenge politically motivated policies where there is evidence of discriminatory and inequitable provision of care, and risk of harm (Nellums et al., 2018a, 2018b). The clear evidence of the deterrent effect of charging and data sharing, the inconsistencies in knowledge around or application of charging policies, and inappropriate charging even where care should not be withheld or should be free, is even more critical now in the context of COVID-19. Whilst there have been statements from government leaders and Public Health England that testing and treatment for COVID-19 is free regardless of legal status, the findings of this research bring attention to the deeper-seated barriers that are likely to prevent equitable access to care and prevention measures such as testing and vaccination, impacting on both individual and public health outcomes.

### 4.3 Strengths and limitations

There have been numerous calls for evidence of the impact of charging and data sharing policies, which have been implemented without a sufficient impact assessment, and not transparently evaluated (O’Donnell et al., 2019; Pike, 2019). This study represents vital data documenting the lived experience of a diverse group of undocumented migrant women affected by these policies. However, there are key limitations which should be acknowledged. In the first instance, the research strengthens our understandings of how immigration status intersects with gender, financial status, and access to healthcare, and women's experiences of marginalisation in the context of these factors. However, as the primary aim of the research was to explore women's experiences pregnancy, we didn't examine women's immigration trajectories in depth, for example, what legal statuses they had previously had, or reasons for being undocumented at the time of interview. Future research could explore with greater detail how different experiences of the legal system further impacted on women's health-related experiences in the UK.

13 participants did not speak English as a first language. Whilst the study utilised appropriate methods for cross-language research (Squires, 2009), translation involves an additional level of interpretation, and the findings are informed by the lived experience not only of the participant, but also the interpreter and the researcher. In addition, we utilised a medical interpreter service. As a result, we worked with several different interpreters, whose approaches varied. For example, in some of the excerpts it can be seen that the interpreter translated in the third person rather than in the first person, though all interpreting was simultaneous. However, we determined that the trustworthiness of the research would be strengthened by using one interviewer and simultaneous interpreting with trained interpreters, rather than multiple interviewers using different languages.

It is important to reflect on the influence the researcher's background may have had on the research. The interviews were conducted by JP, who identifies as Black Caribbean, and has an academic background. She was also not previously known to the participants. Whilst this had the potential to be a limitation as no previous trust or rapport had been established, it may also have enabled women to speak more freely about their experiences, as the researcher was not a member of their clinical or support team, had not known them previously, and they would also be unlikely to interact with her in the future. The researcher's ethnic background may also have influenced her interaction with participants. Whilst she is not a migrant herself, she shares experiences of being an ethnic minority woman the UK.

Barriers to participation may be similar to those experienced in accessing care, and individuals who are more vulnerable or experience the greatest barriers to care may be underrepresented. Thus, the challenges in accessing care, and detrimental impact of charging and data sharing policies may be even more significant than what is reported here. Furthermore, as participants were recruited through DOTW, it is likely that undocumented migrants across England who have not had such support, and experienced even greater difficulties in accessing care are not represented. However, in the interviews themselves, we did not perceive that there were significant barriers which prevented women from being able to engage with the interviewer, or to speak freely about their experiences. The key topics in the topic guide were discussed across all interviews, which may have been supported by the availability of interpreters, the experience of the research team in work with migrant populations, our engagement with Doctors of the World, and the research being carried out in a private, safe, and familiar environment with support resources to hand. It is important to reflect on the implications of Doctors of the World's involvement in the research. Whilst we felt that recruiting participants and ensuring the research did not pose a risk to them was supported by engagement with a trusted organisation focused on supporting their mental, physical, and social needs, we also wanted women to be able to elect or decline to participate confidentially, and the research team's information was provided so that we could be contacted directly where preferred.

## 5 Conclusions

This research highlights the significant risks the charging and data sharing regulations present, and the adverse and inequitable outcomes this is likely to have for maternal and child health. Furthermore, in the context of COVID-19, the research underscores the significant risks posed not only to individual health, but also the wider public health, in deterring access to healthcare for any population.

On the basis of this research and a growing body of evidence (Bragg et al., 2019; Equality and Human Rights Commission, 2019), we have identified six recommendations for immediate implementation in health policy and services, which are vital not only for reducing perinatal morbidity and mortality, but also protecting the wider public health in the context of this pandemic and going forward:

1) Prioritise maternal and child health outcomes, both ensuring timely and appropriate maternity care - which is urgent immediately necessary - is received, and that safeguarding measures for women and babies are made a priority;
2) Suspend charging and all data sharing, including the referral of patients who have incurred a debt of £500 to the Home Office;
3) Ensure entitlement to primary care is respected, and registration is not refused on the basis of immigration status or documentation, in line with the UK Government's commitments to Universal Health Coverage;
4) Ensure adequate information about entitlement to care and data sharing is provided in an accessible and timely manner to both health service staff and patients;
5) Establish fair and reasonable criteria for ability and strategy to pay among chargeable patients; and
6) Ensure policies are evidence-based by improving data collection and monitoring of the accessibility of health services, including for migrant and other marginalised groups, increasing transparency around the impact of any policies on disparities in access to care and health outcomes, and strengthening mechanisms to address such disparities.

The pandemic has underscored the urgency and relevance of these recommendations, and the need to change legislation, both to support public health in the context of this pandemic, and more broadly the right to health for all.

In light of our commitments to conventions such as the United Nations Convention on the Rights of the Child, CEDAW, the Sustainable Development Goals, and our recent adoption of the United Nations Global Compact for Migration, there is a clear need to ensure our health policies are equitable, non-discriminatory, and recognise the right to health. Achieving this, facilitated by these six key recommendations, will be essential to ensure we do not leave migrants behind.

## Author statements

LBN and SH conceived and set up the study with input from LJ; JP, LBN, and KR contributed to data collection and analysis. JP and LBN wrote the first draft, all authors contributed to writing/revisions.
